# Anthocyanin Pigments: Beyond Aesthetics

**DOI:** 10.3390/molecules25235500

**Published:** 2020-11-24

**Authors:** Bindhu Alappat, Jayaraj Alappat

**Affiliations:** Warde Academic Center, St. Xavier University, 3700 W 103rd St, Chicago, IL 60655, USA; alappatjayaraj@gmail.com

**Keywords:** anthocyanins, anthocyanidins, biogenetics, polyphenols, flavonoids, plant pigments, anthocyanin bioactivities

## Abstract

Anthocyanins are polyphenol compounds that render various hues of pink, red, purple, and blue in flowers, vegetables, and fruits. Anthocyanins also play significant roles in plant propagation, ecophysiology, and plant defense mechanisms. Structurally, anthocyanins are anthocyanidins modified by sugars and acyl acids. Anthocyanin colors are susceptible to pH, light, temperatures, and metal ions. The stability of anthocyanins is controlled by various factors, including inter and intramolecular complexations. Chromatographic and spectrometric methods have been extensively used for the extraction, isolation, and identification of anthocyanins. Anthocyanins play a major role in the pharmaceutical; nutraceutical; and food coloring, flavoring, and preserving industries. Research in these areas has not satisfied the urge for natural and sustainable colors and supplemental products. The lability of anthocyanins under various formulated conditions is the primary reason for this delay. New gene editing technologies to modify anthocyanin structures in vivo and the structural modification of anthocyanin via semi-synthetic methods offer new opportunities in this area. This review focusses on the biogenetics of anthocyanins; their colors, structural modifications, and stability; their various applications in human health and welfare; and advances in the field.

## 1. Introduction

**Figure 1:**
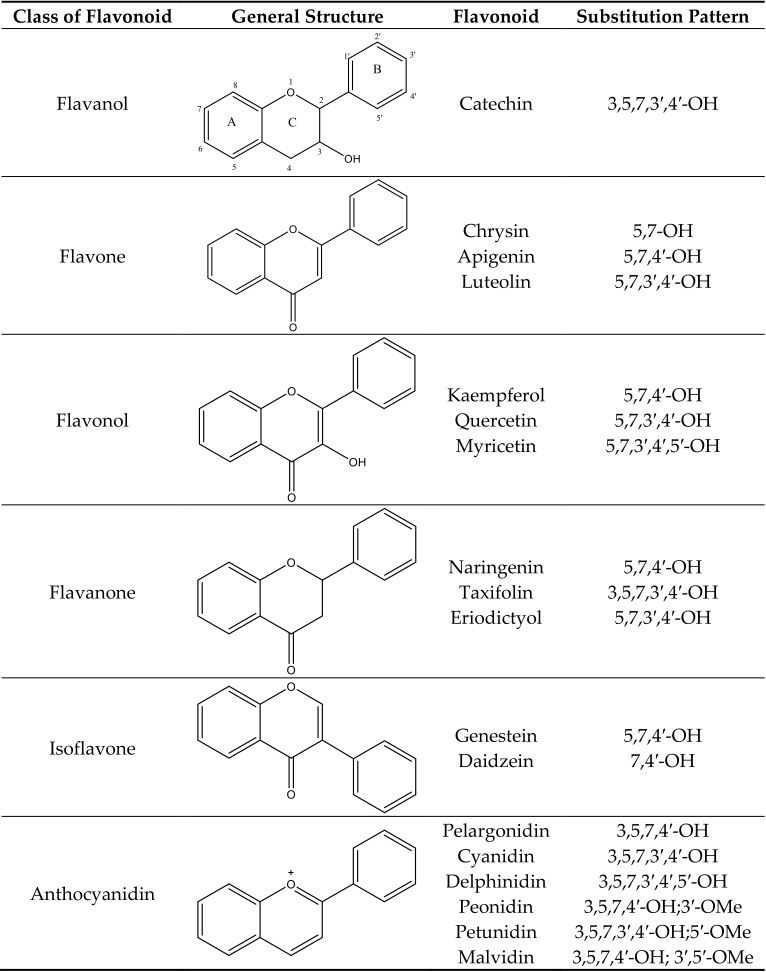
Classification of various flavonoids, their structures and substitution patterns. The ring system (A, B, and C) and numbering is shown for the flavanol structure only. Other structures have the same letters for rings and same numbers for carbon atoms.

Anthocyanins are water soluble pigments that occur in most vascular plants. Anthocyanin is a subgroup of large secondary plant metabolites called flavonoids [1]. More than 5000 flavonoids have been identified. Flavonoids have two aromatic rings connected by a central C3 pyran ring. Common flavonoids are anthocyanins, aurones, chalcones, yellow flavanols, flavones, uncolored flavanols, flavanones, dihydroflavonols, dihydrochalcones, leucoanthocyanidins, catechins, flavans, and isoflavonoids (Figure 1). Anthocyanins are modified by hydroxylation, methylation, glycosylation, and acylation. This adds versatility to the colors and stability of anthocyanins. As the number of hydroxyl groups in the B-ring increases, the color of the anthocyanin becomes bluer. Methylation, on the other hand, leads to a red shift in the color of anthocyanins. Methylation of the B-ring leads to a low susceptibility to oxidation and stabilization of the anthocyanins. Methyl-modified flavonoids are often found in the surfaces of leaves and flowers [2]. Glycosylation of anthocyanins leads to a hypsochromic shift in the absorption maxima of the spectra and increases its stability for storage in the vacuoles [3,4]. The glycosyl moieties of anthocyanins may be further modified by aromatic (hydroxycinnamic or hydroxybenzoic acid) and/or aliphatic (malonic, acetic, or succinic acid) acyl moieties. While aliphatic acylation does not lead to a change in color, aromatic acylation leads to a shift towards blue. Acylation also increases the stability and solubility of anthocyanins [4].

## 2. Biosynthesis of Anthocyanins

**Figure 2:**
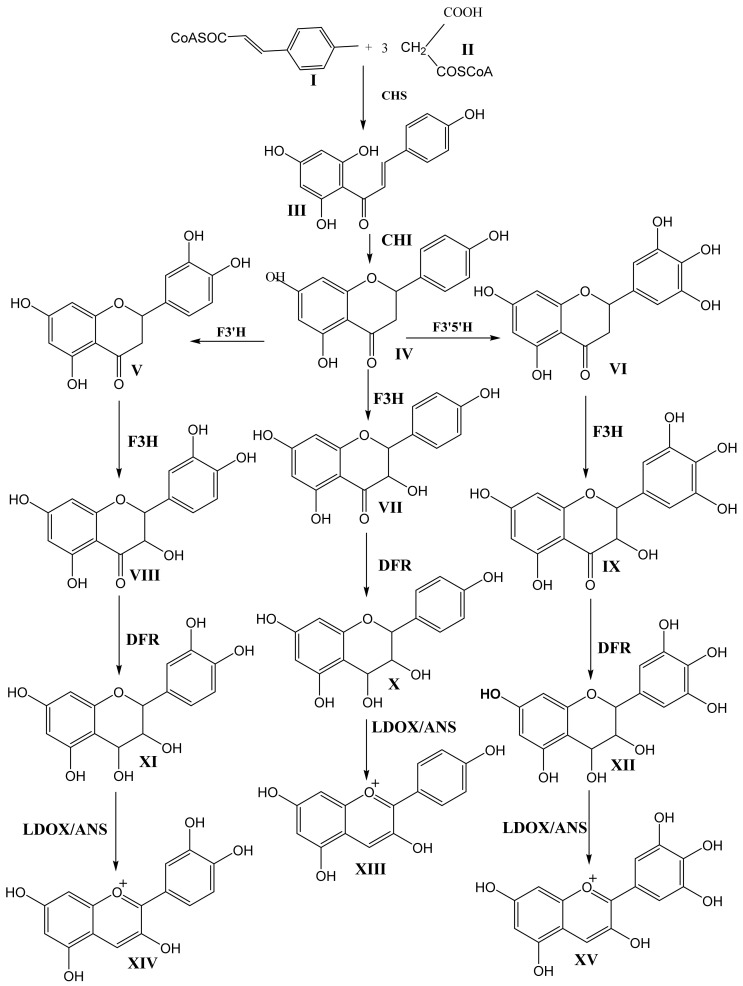
Schematic presentation of the biosynthesis of anthocyanins. CHS, chalcone synthase; CHI, chalcone isomerase; F3H, flavanone 3-hydroxylase; F3′H, flavanone 3′-hydroxylase; F3′5 H, flavanone 3′,5′-hydroxylase; DFR, dihydroflavonol 4-reductase; LDOX/ANS, leucoanthocyanidin dioxygenase/ anthocyanidin synthase [6,7].

**Figure 3:**
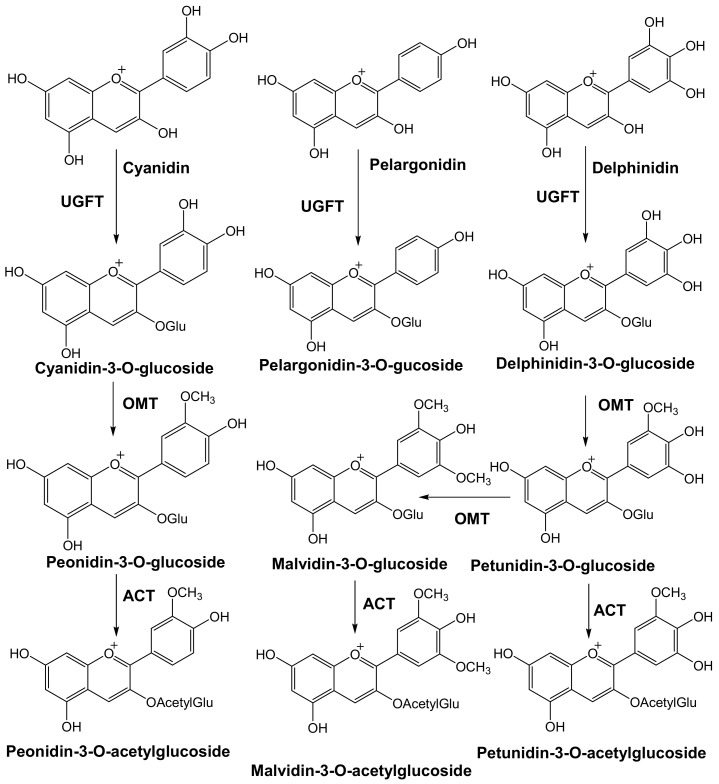
Modification of Anthocyanidins [6] by glycosylation, methylation, and acylation. UFGT, flavonoid glucosyltransferase (UDP-glucose:flavonoid-3-O-gucosyltransferase); OMT, O-methyl transferase; ACT, anthocyanin acyltransferase.

Figure 2 and Figure 3 show the biosynthetic pathway of anthocyanins [1,4,5,6,7]. This pathway is well understood and is conserved among seed plants. The organization of enzymes involved in the pathway into various classes (like 2-oxoglutarate-dependant dioxygenase, OGD, and cytochrome P450) suggests that plants recruit these enzymes from pre-existing metabolic pathways [4]. Previous studies have shown that the enzymes earlier in the pathway are encoded in large gene families, whereas enzymes acting later in the pathway are commonly encoded in single active genes [6].

The first committed step in the pathway is the condensation of one molecule of p-coumaryl-CoA (I) with three molecules of malonyl-CoA (II) via the action of chalcone synthase (CHS) to produce tetrahydroxy chalcone (III). Isomerization of tetrahydroxy chalcone through the action of chalcone isomerase (CHI) produces the flavanone naringenin (IV). Naringenin acts as the central branch point of various flavonoids through hydroxylation on the C-ring via flavanone-3-hydroxylase (F3H) and hydroxylation on the B-ring via flavanone 3′-hyroxylase (F3′H) or flavanone 3′5′-hydroxylase (F3′5′H) to produce dihydrokaempferol (VII), dihydroquercetin (VIII), and dihydromyricetin (IX). F3H belongs to the OGD family, and both F3′H and F3′5′H belong to P450. The B-ring hydroxylation of naringenin by flavanone 3-hydroxylase and flavanone 3′5′-hydroxylase produces eriodictyol (V) and pentahydroxy flavanone (VI), respectively. These hydroxylation enzymes are necessary for the production of cyanidin (XIV) and delphinidin (XV). The absence of F3′5′H is attributed to the lack of violet/blue colors in several floricultural crops, such as roses (*Rosa hybrida*) and chrysanthemums (*Chrysanthemum morifolium*), due to their inability to produce delphinidin. However, transgenic blue/violet roses have been developed [4].

The next step in the biosynthesis of anthocyanin is the action of Dihydroflavonol 4-Reductase (DFR) on one of the three didydroflavonols, dihydrokaempferol (VII), dihydroquercetin (VIII), or dihydromyricetin (IX), to produce the corresponding leucoanthocyanidins leucopelargonidin (X), leucocyanidin (XI), and leucodelphinidin (XII). DFR has strict substrate specificity. For example, in petunia (*Petunia hybrida*) and cymbidium (*Cymbidium hybrida*), DFR cannot use dihydrokaempferol as the substrate. These plant species, therefore, lack pelargonidin-based anthocyanin colors like orange and brick red [4]. Leucoanthocyanidins are converted into the corresponding anthocyanidins (orange-colored pelargonidin (XIII), orange-red-colored cyanidin (XIV), and bluish-red (XV) delphinidin) by the action of leucoanthocyanidin dioxygenase/anthocyanidin synthase (LDOX/ANS). LDOX/ANS belongs to the OGD family.

**Figure 4:**
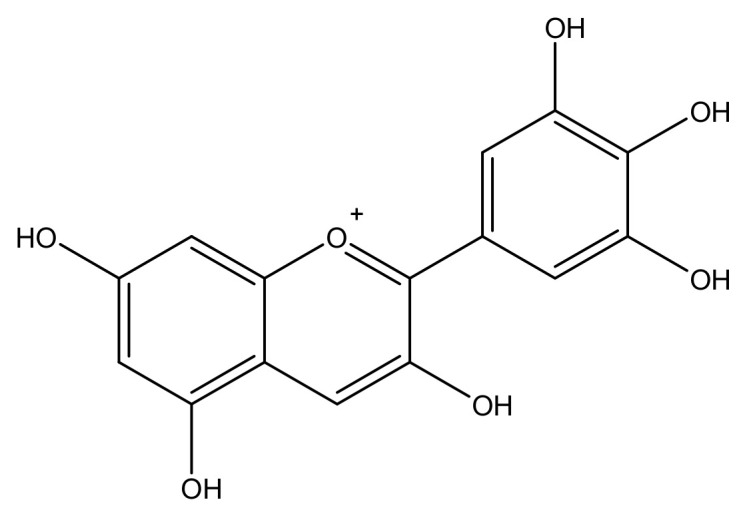
Structure of a flavylium cation from delphinidin.

**Figure 5:**
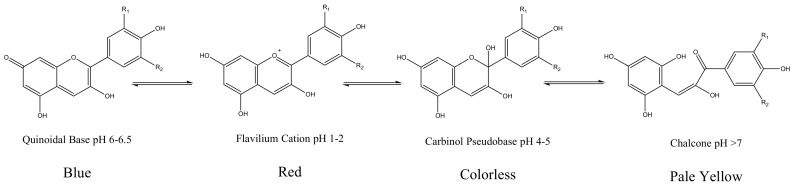
Various conformations of delphinidin and their colors in solution with various pH conditions [5]. R_1_ and R_2_ represent the -OH and -OMe groups in various anthocyanidins.

Anthocyanidin are most commonly presented as flavylium cations (Figure 4). The colored flavylium cations are stabilized inside the cell by intra and intermolecular complexation. Intermolecular complexations are facilitated by colorless flavonols, carotenoids, or metal ions like Mg^2+^ or Al^3+^ [7]. These modifications, along with the stacking of planar anthocyanins, render increased stability and add versatility to anthocyanin’s colors. However, anthocyanins are prone to changes in color and conformation in solutions at various pH values, as presented in Figure 5 [5].

Naturally occurring types of anthocyanidin produced via substitution at various position in the flavylium cations are shown in Table 1 [3]. The most abundant anthocyanidins in nature are pelargonidin, cyanidin, delphinidin, peonidin, petunidin, and malvidin. Hydroxylation and methylation of the B-ring control the color and the stability of anthocyanins. Blueness is increased with an increased number of hydroxyl groups, while redness increases with enhanced methylation in the B-ring. O-methyl transferases, moreover, mediate the methylation of hydroxyl groups on the B-ring.

The presence of Ortho hydroxyl groups in the B-ring makes the molecule more susceptible to oxidation, thereby reducing the stability of the molecule. The ortho-positioned hydroxyl groups in cyanidin, delphinidin, and petunidin make them less stable than peonidin and malvidin [6].

The next step in the biosynthesis of anthocyanins is O-glycosylation. In contrast to the well-conserved main flavonoid pathway, the modification of anthocyanidin by glycosylation is diverse, with family and species dependency. The enzymes driving these modifications are specific to the position of the modification and the donor substrate. Glycosylation is facilitated by glycosyl transferases found in the cytoplasm. Anthocyanins are initially glycosylated in the 3 position. However, in studies where 3,5 diglucosides were accumulated, 5-glucosylation preceded 3-glucosylation [4].

**Figure 6:**
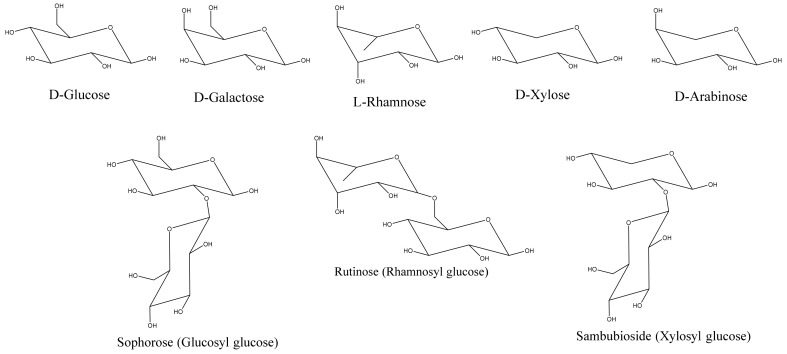
Common mononsaccharides and biosides that modify anthocyanins.

Common sugars that modify/decorate anthocyanins are given in Figure 6. Glycosylation occurs immediately after the action of ANS to stabilize anthocyanidins [5,8,9,10,11]. C3, C5, C7, C3′, and C5′ are all accessible to glycosylation. C3 glycosylation is the most common among the naturally occurring anthocyanins. The most commonly studied glycosylation involves the addition of the glucose group via UDP-3-O-glucosyltransferases (UFGT/3GT). C3 glycosylation leads to a red shift in the color of the anthocyanin with increased stability. C3 biosides, moreover, are more stable than monoglycosides, and 3,5 diglycosides are also common in the plant kingdom. While 3 glycosides increase stability, 5 glycosides tend to reduce stability. Moreover, decorating anthocyanidin with 5-glycosides could also lead to the formation of colorless pseudobases because the loss of the hydroxyl group in position 5 makes the anthocyanin more susceptible to a hydration reaction.

**Figure 7:**
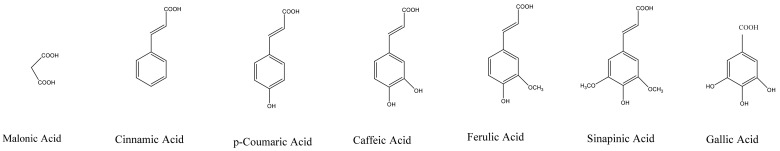
Common acids involved in the acylation of anthocyanins.

Sugar residues in anthocyanins are often acylated by aromatic or aliphatic acids. Common aliphatic and aromatic acids involved in acylation reactions are given in Figure 7. Acylation leads to a change in color (blue shift) and increased stability due to intra and intermolecular co-pigmentation reactions [5]. In addition to the biosynthetic genes involved in the formation of anthocyanin pigments, vacuolar pH and cell shape have a dramatic effect on anthocyanin pigments [7]. In petunia flowers, acidification of the vacuole changes the color to red, and mutations affecting the pH manifest with changes in the color towards blue. Even with a high accumulation of pigments, plants may appear color-deficient due to the shapes of their cells. This is because of the differences in reflected light between conical and flat cells [7].

Flavonoid glycosides are usually transported into the vacuoles. The mechanism by which this occurs, however, is not well understood. Transportation of anthocyanins from the sites of synthesis to the vacuoles is induced by the action of several transporters, including glutathione S-transferase (GST), multidrug and toxic compound extrusion (MATE) transporters, and multidrug resistance-associated protein (MRP) transporters [8,12,13]. The final color of anthocyanin is controlled by the pH of the vacuole. Structural genes that regulate the pH in Japanese morning glory and petunia have also been identified [4].

## 3. Extraction, Isolation, and Identification of Anthocyanins

Anthocyanins are polar in nature. The use of polar solvents like methanol and ethanol makes extraction of anthocyanins efficient. However, for food grade applications, ethanol is preferred over methanol. Often, organic (acetic, citric, or tartaric acids) or mineral acids (hydrochloric acid or phosphoric acid) are added to the extraction solvent to stabilize the flavylium cation. The use of hydrochloric acid is restricted, as hydrochloric acid might break down acylated anthocyanins [1,14]. The temperature of the extraction also affects the recovery of anthocyanins. Extraction processes are repeated for several rounds, depending on the extractability of the system, as well as the quantities of total anthocyanins present in the matrix. For commercial applications, the extracts are concentrated into a thick paste under a reduced vacuum in a rotavapor. The concentrated extract is freeze-dried to obtain a fine anthocyanin powder.

Nicoue et al. compared the effects of the temperature, solvent, and pH of the extraction solvent on the recovery of anthocyanins in wild blueberries [15]. Extraction in ethanol with phosphoric acid (pH, 4.6; 0.02% *v*/*v*) gave the highest yield of total and monomeric anthocyanins. Anthocyanins are separated from sugars and other organic compounds by clarification using C18 cartridges. In principle, anthocyanins will be absorbed (loaded) onto the cartridge, and all non/less-absorbed materials will be washed away with 0.01% HCl in water. Pure anthocyanins will later be eluted with 0.01% HCl in methanol [15]. Due to the number of modifications of anthocyanin molecules by glycosylation and acylation, the analysis of anthocyanin mixtures is cumbersome. There are several ways to reduce this complexity. Acid hydrolysis is one of the most preferred methods. Anthocyanins are easily hydrolyzed with 2N HCl by heating at 100 °C. Anthocyanidins with cleaved carbohydrates can then be purified as in anthocyanin purification.

UV/Vis spectrophotometric methods and HPLC methods using anthocyanin standards are the methods of choice for anthocyanin identification and quantification [15,16]. Many anthocyanin pigments have characteristic absorption profiles in the UV-Vis spectral region, which makes UV-Vis an invaluable tool for the identification of molecules with B-ring substitution [17].

## 4. HPLC Analysis of Anthocyanins

HPLC analysis has been the method of choice for the detection and quantification of anthocyanins for the past fifty years [17,18,19,20]. The discovery of reverse phase columns that are able to separate anthocyanins and photodiode array detection (PDA) added versatility to this technique. Normal conditions in HPLC are C18 columns; a detection wavelength of 520 mm; acetonitrile: and Water solvent systems with pH adjusted by phosphoric acid, formic acid, or trifluoro acetic acid. The sensitivity of HPLC techniques, along with their simplicity, has made this method of analysis popular in qualitative and quantitative studies of anthocyanins and anthocyanidins. The spectra generated by PDA detection in hyphenated HPLC-PDA systems make the identification of anthocyanin compounds easier. Three regions in the spectra are evaluated. The absorption characteristics at 310 nm compared to those at 510–550 nm provides information on the acylation of anthocyanins with hydroxylated aromatic acids [17]. The absorption characteristics at 440 nm show the differences between 3-glycosides and 3,5-diglycosides. 3-glucosides will normally have double the absorbance of 3,5-diglycosides [17]. There are more than 250 naturally occurring anthocyanins formed from the glycosylation of six common anthocyanidins with various sugar molecules. The non-availability of standards is one of the biggest challenges for chromatographers. The acid hydrolysis of anthocyanin extracts significantly simplifies the HPLC chromatograms for common anthocyanidins. These simplified chromatograms and the availability of standard anthocyanins make anthocyanin quantification accurate [21]. In complex samples where the separation of individual components is low, two-dimensional liquid chromatography (LCXLC) has been applied for the identification of molecules [22].

## 5. Structure and Stability of Anthocyanins

Anthocyanins are susceptible to temperature, pH, and light [23,24]. Anthocyanins change their color to yellow or colorless degradation products under these conditions. Several mechanisms have been proposed to ensure the stability of anthocyanins. The degradation and metabolism of anthocyanin molecules may occur in the A or B ring of the molecules. The metabolism of anthocyanins has also been studied extensively [25,26,27]. It was observed that the substitution of hydroxyl groups of the B-ring by the methoxy group increased the stability of anthocyanins in simulated gastric digestion processes. Syringic acid, protocatechuic acid, and vanillic acid were the main degradation products under these conditions [24].

The possibility of anthocyanins to be hydrated has been attributed to the loss of color in anthocyanins. Various color stabilization mechanisms, including metal ion complexation, the complex formation of anthocyanins with flavonoid co-pigments (co-pigmentation), self-association, and intramolecular sandwich-type stacking of the anthocyanin nucleus and acyl groups have all been explored [23].

## 6. Nutraceuticals and Applications of Anthocyanins

Anthocyanins are emerging as one of the most promising ingredients in the food, beverage, cosmetics, and nutraceutical industries. The nutraceutical activities of anthocyanins and anthocyanidin have been reviewed extensively [14,28,29,30,31,32]. Anthocyanins have also been used traditionally as a natural food coloring agent.

Numerous studies have revealed the antimicrobial activities of anthocyanins [33,34,35]. The antimicrobial activities of anthocyanin are attributed to the destruction of the cell wall, cell membrane, and intercellular matrix. Antimicrobial activity was also attributed to the ability of anthocyanins to release polysaccharide molecules from the outer membrane of Gram-negative bacteria. Anthocyanins may also affect microbial metabolism by depriving the organism of substrates required for its growth [33].

The anticancer activities of anthocyanin have also been studied and reviewed extensively [36]. Nichenametla et al. reviewed the anticancer actions and mechanisms of anthocyanins [32]. The effect of Cyanidin-3-Glucoside (C3G) to block the ethanol-induced activation of the ErbB2/cSrc/FAK pathway was studied by Xu et al. [37]. The ability of C3G to prevent cell migration/invasion was touted as beneficial to prevent ethanol-induced breast cancer metastasis. The abilities of anthocyanins to induce apoptosis and suppress angiogenesis were explained as the reasons for the anticancer activities of anthocyanins [38]. Bontempo et al. studied the anticancer activities of anthocyanins in Vitelotte potato [39]. Yi et al. assessed the effect of anthocyanin in muscadine grapes on cancer cell viability and apoptosis [40]. Anthocyanins in purple tea exhibited antioxidant, immunostimulatory, and anticancer activities [41].

Diabetes mellitus (DM) is a chronic metabolic disorder affecting millions of adults between 20 to 79 years of age. The number of people afflicted by DM has quadrupled in the past three decades, with type-2 DM comprising 90% of DM cases. DM is thus proving to be a global health concern [42]. This metabolic disorder is characterized by increased sugar concentration in the blood cause by impaired insulin secretion or insulin resistance. The affective management of diabetes, therefore, is to prevent excess postprandial increases in the blood glucose level and improve insulin resistance. The antidiabetic activities of anthocyanins have been studied extensively [43,44,45,46,47,48,49,50,51,52,53]. Acylated anthocyanin petunidin-3-O-p-coumaryl-rutinoside-5-O-glucoside, which is the main anthocyanin component in the extract from the Blue Congo variety of purple potato, reduced the fasting sugar levels in streptozotocin-induced diabetic rats [43]. The anthocyanin extract from mulberry fruit exhibited significant antidiabetic properties by reducing glucose levels in Zucker Diabetic Fatty rats [44]. Belwal et al. [45] reviewed the role of anthocyanins in ameliorating insulin resistance—an abnormal physiological state where insulin from pancreatic β-cells is unable to trigger a signal transduction pathway in the target organs. Cyanidin stimulated insulin secretion and pancreatic β-cell gene expression. Cyanidin also up-regulated the expression of the genes that have potential implications on insulin secretion, glucose homeostasis, and diabetes [46]. Purple corn anthocyanins increased insulin secretion in HIT-T15 cells (ATCC CRL-1777, hamster pancreatic beta cell line) and db/db mice [48]. The ability of acetylated anthocyanins to decrease postprandial glucose levels through retarding maltase activity was investigated by Matsui et al. [52].

## 7. Anthocyanins as Food Coloring Agents

Due to the dynamically growing natural, organic, and sustainable food markets, the demand for non-synthetic food colorants continues to increase. Anthocyanins have filled this gap for the last three decades. Anthocyanins have additional health benefits that give them a unique position among anthocyanins in this sector. Anthocyanins have been studied for their application as food colorants due their vibrant colors [29,30,53,54,55,56,57]. Anthocyanins have also been used in various applications to render pink, red, purple, and blue colors. Because of their high reactivity and destabilizing interactions with other molecules in the media, the application of anthocyanins as food colorants has been limited. Co-pigmentation, complexation with various metal ions, and acylation with various organic acids have all been attempted to increase the stability of anthocyanins. Polyphenol oxidase is one of the enzymes that has been identified as the reason for the color destabilization of anthocyanins [53]. The application of anthocyanin to produce various colors in a pH range of 2–8 was reported using a pelargonidin-based anthocyanin extracted and purified from the flowers of *Ipomea tricolor* [53]. Companies that produce anthocyanin-based natural colors, their products, and their strategies to stabilize anthocyanins in food applications were reviewed by Cortez et al. [57].

## 8. Anthocyanins as Antioxidant and Anti-inflammatory Agents

The demand for natural antioxidants is on the rise. The urge to shift from synthetic to natural products has been driven by the research in this area [58,59,60,61,62,63,64,65,66,67,68,69,70,71]. Several methods have been used for the evaluation for antioxidant capacities of polyphenols in general and anthocyanins in particular. TEAC (Trolox Equivalent Antioxidant Capacity), FRAP (Ferric Reducing Ability of Plasma), and ORAC (Oxygen Radical Absorbance Capacity) assays are only a few among the many popular assays deployed by the scientific community [58]. Table 2 outlines the antioxidant capacities of common fruits and vegetables containing anthocyanins. In general, antioxidant capacities are correlated with polyphenolic content [58]. Many members of the anthocyanin family demonstrated antioxidant activities similar to α-tocopherol, Trilox, Quercetin, and Catechin. Table 2 outlines the experimental antioxidant capacities of common fruits and vegetables along with their polyphenolic content, calculated as Gallic Acid Equivalents [58], while Table 3 outlines the Antioxidant capacities (TEAC) of some common anthocyanins and anthocyanidins [67].

The antioxidant efficacy of anthocyanins is controlled by their structures. The three-dimensional Quantitative Structure Activity Relationships (3D-QSAR) of anthocyanins extracted from eggplant and radish on the Oxygen Radical Absorbing Capacity were studied by Jing et al. [67]. The effects of hydroxyl groups on the B-ring of anthocyanidin, methoxylation, and the number of glycosyl units in in position 3 were instigated by Seeram et al. [59]. The substitution of hydroxyl groups with methoxy groups in the B-ring decreased antioxidant capacity. Anthocyanins were able to act as reducing agents in the electron-transfer reaction pathway with the ability to donate electrons to the free radicals with unpaired electrons [68]. Anthocyanins are some of the strongest antioxidants due to their free radical scavenging abilities. Two free radical scavenging pathways are possible due to the hydroxyl groups in the B-ring, as well as the oxonium ion in the C-ring [68,69].

Studies on the anti-inflammatory activities of anthocyanins may be the next well-explored area of anthocyanin research [64,66,72,73,74,75,76,77] for human health and wellness. The ability of anthocyanins to inhibit lipoxygenase and cyclooxygenase 2 enzymes has also been studied [64]. Obesity is a metabolic syndrome occurring worldwide and is often associated with other chronic diseases, such as cardiovascular disorders, type II diabetes, and cancer. Inflammation play a pivotal role in the pathogenesis of obesity. The role of anthocyanins in ameliorating obesity and obesity-associated disease was reviewed by Lee et al. [72]. Ulcerative colitis (UC), which is a major form of inflammatory bowel disease (IBD), is a chronic relapsing disorder. Several studies demonstrated [74,76] the anti-inflammatory properties of anthocyanins and the potential of anthocyanins to be used as novel therapeutic agents in UC treatment.

## 9. Advances in Anthocyanin Research

The increased use of anthocyanins in various industries and their potential health benefits have led to several studies exploring efficient, economical, and nature-friendly extraction techniques. The use of organic solvents in the extraction and purification of anthocyanins has been scrutinized due to their environmental and undesirable biological impacts. Intensive research has focused on sustainable extraction procedures. Green extraction techniques like the use of supercritical fluids (SCFs) have gained momentum in the past few years due to their benefits for the environment [78].

Anthocyanins are a class of pigment that have been studied extensively for their biogenetics [79,80,81,82,83]. Prior to the validated studies detailing their many bioactivities, research focused on the various genes in the pathway for the modification and stabilization of colors. Molecular modifications for stabilizing anthocyanin colors for nutraceutical and food colorant applications have resulted in the use of molecular biology techniques to develop new products that address the demands of the food, beverage, and nutraceutical markets. The growing demand for natural alternatives to food colorants and the increased awareness of the environmental hazards of synthetic analogues have accelerated research initiatives towards this goal. The global demand for natural colorants has increased in the past decade [84]. This had forced several food colorant companies to shift to natural food colorants. The cost associated with creating a stable natural color, however, had been the greatest challenge facing this industry. This issue has prompted the search for novel and economically viable solutions for the production, extraction, purification, and stabilization of anthocyanin-based food colorants. The shift from synthetic to natural colorants depends on the stability of these colors in various food matrices and formulated conditions. The observed stability of acylated and co-pigmented anthocyanins has opened up new opportunities for food producers and color formulators [85,86]. The ability of these pigments to maintain colors under various pH values and processing conditions makes them suitable candidates for dairy and ready-to-eat desserts. Stabilizing methods also include the addition of compounds, such as polymers, phenolic compounds, and metals. The exclusion of oxygen during processing and storage and the encapsulation of pigments have also been considered as successful techniques for the stabilization of anthocyanin colors. Combining synthetic and semisynthetic methods along with formulation techniques using new materials capable of stabilizing anthocyanins will enhance the potential of anthocyanins for use as value-added natural food pigments [57].

## 10. Conclusions

Anthocyanins, and the pathways leading to the production of myriad members in their family, represent one of the most commonly researched areas in the biogenetics of natural pigments. This focus (detection of molecules, their stability, and existence in various organisms) was driven by the aesthetics associated with these molecules. Consumer preferences for natural food colorants, anthocyanin-based nutraceuticals, and QSAR studies have caused a shift in anthocyanin research. The renewed interest in the past two decades, driven by interests from consumers, food industry, and the scientific community as well as their collaborations has been commendable. Anthocyanins exert antioxidant and anti-inflammatory activities. Their ability to alleviate complications arising from cancer, diabetes, and other metabolic disorders has been validated at the laboratory level. More research in animal and human trials, however, is needed to elucidate the relevant mechanisms of action.

Consumers preferences for natural ingredients are based on the fewer side effects associated with natural ingredients in contrast to synthetic/artificial ingredients. Consumers are also willing to compromise cost as increasingly more people become engaged in global sustainability issues. Industries are now considering taking the best of these products from research initiatives into the market and have been able to address many issues related to storage and stability. The cost of production and a lack of market are two issues currently facing this industry. Consumers also seek convincing data to alter their decisions and thus move from synthetic to natural ingredients. Industries need to translate research data into products and support research in this area. The application of anthocyanin as a natural food colorant is more appealing to the consumer because of the complementary benefits offered by anthocyanin’s base colors.

The structural modification of anthocyanins to enhance their bioactivities is an area that need to be explored further. Research has shown that the acylation of anthocyanins improves their stability. Structural modifications also improve their bioactivities. Developing new anthocyanin-accumulating crops via traditional breeding takes several years. This timeline will not be able to satisfy the demand for developing novel crops faster. Gene editing offers a faster method to develop these crops. New CRISPR-Cas9 (CRISPR for Clustered Regularly Interspaced Short Palindromic Repeats) is a powerful genome editing tool that has recently been widely adopted in model organisms [87,88].

The roles of anthocyanins in ameliorating various ailments have been an integral part of the fabric of human–nature interactions. However, a deeper understanding of the mechanisms and modes of their action leading to these effects have not been elucidated. A concerted effort by academia, industry, and consumer advocacy groups is currently needed to explore the potential of these pigments and develop sustainable products for human health and wellbeing.

## Figures and Tables

**Table 1:**
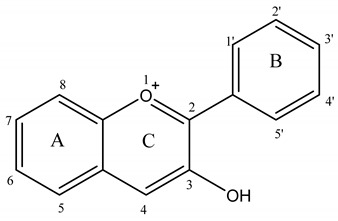
Naturally occurring anthocyanidins produced via substitution of the flavylium cation.

Anthocyanidin	Substitution Pattern in Various Positions							Color
	3	5	6	7	3′	4′	5′	
Carajurin	H	H	OH	OH	H	OCH_3_	OCH_3_	-
Arrabidin	H	H	OH	OH	H	OH	OCH_3_	-
3′-hydroxyarrabidin	H	H	OH	OH	OH	OH	OCH_3_	-
Tricetinidin	H	OH	H	OH	OH	OH	OH	Red
Pelargonidin	OH	OH	H	OH	H	OH	H	Orange
Aurantinidin	OH	OH	OH	OH	H	OH	H	Orange
Cyanidin	OH	OH	H	OH	OH	OH	H	Orange red
5-methyl cyanidin	OH	OCH_3_	H	OH	OH	OH	H	Orange red
Peonidin	OH	OH	H	OH	OCH_3_	OH	H	Red
Rosinidin	OH	OH	H	OCH_3_	OCH_3_	OH	H	Red
6-hydroxycyanidin	OH	OH	OH	OH	OH	OH	H	Red
6-hydroxydelphinidin	OH	OH	OH	OH	OH	OH	OH	Bluish red
Delphinidin	OH	OH	H	OH	OH	OH	OH	Bluish red
Petunidin	OH	OH	H	OH	OCH_3_	OH	OH	Bluish red
Malvidin	OH	OH	H	OH	OCH_3_	OH	OCH_3_	Bluish red
Pulchellidin	OH	OCH_3_	H	OH	OH	OH	OH	Bluish red
Eupinidin	OH	OCH_3_	H	OH	OCH_3_	OH	OH	Bluish red
Capensinidin	OH	OCH_3_	H	OH	OCH_3_	OH	OCH_3_	Bluish red
Hirsutidin	OH	OH	H	OCH_3_	OCH_3_	OH	OCH_3_	Bluish red

**Table 2:** Antioxidant capacities of common fruits and vegetables along with their polyphenolic content calculated as Gallic Acid Equivalents [58].

Fruit/Vegetable	TEAC (mmol Trolox/100 g FW)	FRAP (mmol Fe^2+/^100 g FW)	ORAC (mmol Trolox/100 g FW)	Total Phenolics (mg GAE/100 g FW)
Strawberry	2591 ± 68	3352 ± 38	2437 ± 95	330 ± 4
Red Plum	1825 ± 28	2057 ± 25	2564 ± 185	320 ± 12
Red Cabbage	1377 ± 49	1870 ± 18	2124 ± 68	158 ± 4
Onion	532 ± 29	369 ± 13	988 ± 30	88 ± 1
Pea	440 ± 18	251 ± 9	704 ± 62	32 ± 1
Apple	434 ± 13	394 ± 8	560 ± 18	48 ± 1
Tomato	255 ± 14	344 ± 7	420 ± 39	30 ± 1

**Table 3:**
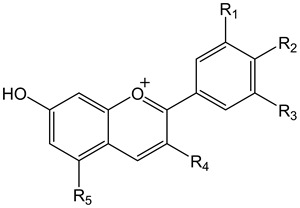
Antioxidant capacities (Experimental TEAC) of some common anthocyanins and anthocyanidins [67].

R_1_	R_2_	R_3_	R_4_	R_5_	Experimental TEAC
OH	OH	H	OH	OH	6.764
H	OH	H	OH	OH	5.801
OH	OH	OH	OH	OH	3.084
OH	OH	H	O-glc	OH	4.723
H	OH	H	O-glc	OH	6.309
OH	OH	OH	O-glc	OH	5.137
OH	OH	H	O-gal	OH	9.608
OH	OH	H	O-ara	OH	1.286
OH	OH	H	O-rut	OH	5.940
OH	OH	H	O-sop	OH	5.809
OH	OH	OH	O-rut	OH	4.098
OH	OH	H	O-glc	O-glc	4.468
H	OH	H	O-glc	O-glc	5.808
OCH_3_	OH	H	O-glc	OH	5.178
OCH_3_	OH	H	O-gal	OH	2.615
OCH_3_	OH	OCH_3_	O-glc	OH	2.916
OCH_3_	OH	H	O-ara	OH	4.070
OCH_3_	OH	OCH_3_	O-glc	O-glc	4.099

glc = glucose; gal = galactose; ara = arabinose; rut = rutinose; sop = sophorose.
